# Comparative Proteomic Analysis of Milk-Derived Extracellular Vesicles from Dairy Cows with Clinical and Subclinical Mastitis

**DOI:** 10.3390/ani13010171

**Published:** 2023-01-01

**Authors:** Mengling Wang, Meng Cai, Xiaoyan Zhu, Xuemei Nan, Benhai Xiong, Liang Yang

**Affiliations:** State Key Laboratory of Animal Nutrition, Institute of Animal Science, Chinese Academy of Agricultural Sciences, Beijing 100193, China

**Keywords:** milk, extracellular vesicles, proteome, cow, mastitis

## Abstract

**Simple Summary:**

Extracellular vesicles in milk may play important roles in the development of clinical and subclinical mastitis. Milk-derived extracellular vesicles of mastitis and healthy cows were extracted by a combination method of acetic acid/ultracentrifugation and density gradient ultracentrifugation and analyzed using a Shotgun proteomic by data-independent acquisition mode. Our data indicated that mammary inflammation altered milk-derived EV (Extracellular vesicle) protein cargos, especially antimicrobial peptides. These results expanded the repertoire of bovine milk-derived extracellular vesicles proteins and conduced more attention to the functions and the pathways they might impact.

**Abstract:**

Extracellular vesicles (EVs) are membranous vesicles found in biological fluids with essential functions. However, milk-derived EV proteins from clinical mastitis (CM) and subclinical mastitis (SM) cows have yet to be studied in detail. In this study, milk-derived EVs of CM, SM, and Healthy cows were extracted using a combination of acetic acid/ultracentrifugation and density gradient ultracentrifugation and analyzed using a shotgun proteomic by data-independent acquisition mode. A total of 1253 milk exosome proteins were identified and quantified. Differently enriched (DE) proteins were identified as given a Benjamini–Hochberg adjusted *p* < 0.05 and a fold change of at least 2. There were 53 and 1 DE proteins in milk-derived EVs from CM and SM cows compared with healthy cows. Protein S100-A9, Protein S100-A8, Chitinase-3-like protein 1, Haptoglobin, Integrin beta-2, and Chloride intracellular channel protein 1 were more abundant in the CM group (adjusted *p* < 0.05). Still, their enrichment in the SM group was not significant as in the Healthy group. The enrichment of DE proteins between CM and Healthy group was consistent with elevated GO (Gene Ontology) processes—defense response, defense response to Gram-positive bacterium, granulocyte chemotaxis also contributed to Reactome pathways—neutrophil degranulation, innate immune system, and antimicrobial peptides in the CM group. These results provide essential information on mastitis-associated proteins in milk-derived EVs and indicate the biological functions of milk-derived EVs proteins require further elucidation.

## 1. Introduction

Most living cells release extracellular vesicles (EVs) during normal or abnormal physiology [1]. The EV family comprises exosomes, microvesicles, apoptotic EVs, and other EVs [2]. EV populations are heterogeneous and can be roughly categorized into two categories, exosomes and ectosomes, based on size and biogenesis [3]. Exosomes are the most studied EV subtype to date, which are generated within the multivesicular bodies (MVB) by endosomal sorting complexes required for transport (ESCRT) complex [4]. Tetraspanins (CD63, CD9, and CD81) [4], ESCRT components (TSG101, VPS4, and Alix), and cytosolic proteins Hsp70 were mainly used to demonstrate the EV subtype purity [3]. Exosomes play an essential role in intercellular communication; they have the ability to deliver their cargo (including proteins, lipids, and nucleic acids) to recipient cells; the crucial role of exosomes is also indicated in many pathophysiological processes [2].

EVs were isolated from all body fluids, including blood, urine, milk, saliva, tears, and ascites [5,6]. Both raw and commercial milk contained EVs enriched with proteins and microRNAs [7]. The phospholipid bilayer of EVs confers protection to the luminal cargo against harsh degrading conditions [8,9]. Milk-derived EVs can be taken up by human and murine intestinal or murine cells in vitro [8,9]. Previous studies demonstrated that bovine milk-derived EVs could regulate immunity, metabolism, growth, and development [10,11,12]. A complete knowledge of the milk EVs proteome is essential in dairy product manufacturing. 

Mastitis, an inflammation of the mammary gland, the most frequent disease of dairy cows, remains the primary cause of economics due to reduced milk yield and quality, increased treatment costs, and cow mortality [13]. Clinical mastitis can be detected by changes in milk, such as the appearance of clots, flakes, or watery texture. Subclinical mastitis is challenging to detect, usually manifested by increases in somatic cell counts (SCC) without any visible clinical symptoms or clinical evidence [14]. The incidence of subclinical mastitis in Brazil’s modern dairy farms is much higher than in clinical mastitis [15]. The main reason for inflammation in mammary glands is bacterial infections, and *Staphylococcus aureus* is the most important contagious pathogen responsible for bovine mastitis [13]. Previous studies have investigated the proteome of colostrum and mature milk-derived EVs [7,16,17], indicating the potential physiological significance of EVs proteins to bovine mammary glands. Proteomic analysis and microRNA expression profiles of bovine milk-derived exosomes infected by *S. aureus* have been widely studied [18,19,20,21]. Chronic subclinical mastitis induced changes in the immunoregulatory miRNAs of milk-derived EVs cargo [22]. To date, few studies have investigated the milk-derived EVs from cows with subclinical mastitis, and more research is needed.

Here, we used a Shotgun proteomic by data-independent acquisition (DIA) approach to push our investigations further and provide a complete comparison of the mastitis-associated milk-derived EVs proteins. This work may help to identify protein markers specific to clinical or subclinical mastitis, provide insights into the function of the proteins they contain, and the pathways they might impact and guide researchers.

## 2. Materials and Methods

### 2.1. Animal Health and Milk Sampling

All experimental designs and protocols in this study were approved by the Animal Ethics Committee of the Chinese Academy of Agricultural Sciences (Beijing, China; approval number: IAS-2020-60). All the experiments were performed according to the academy’s guidelines established by this committee. The study was conducted on a commercial dairy farm in Beijing, China. Milk samples were collected from milking Holstein-Friesian cows during normal lactation. The current research conducted and recorded each cow’s overall condition, the udder’s clinical manifestations, the California mastitis test (CMT), and the SCC of milk. We also performed the pathogens culture with AccuMast detection plates (FERA Animal Health LCC, Ithaca, NY, USA) for cows with clinical mastitis. Cows with apparent signs of other diseases or recently received antibiotic treatment were excluded. Two milk samples were collected from each cow: one milk sample was immediately treated with 0.4% potassium dichromate and analyzed for milk components and SCC by FTS/FCM series instruments (Bentley Instruments, Chaska, MN, USA); another milk sample was stored at −80 °C until use. A threshold of 200,000 cells/mL was used to define a case of subclinical mastitis [15]. A total of 15 cows were selected and diagnosed into 3 groups: 5 healthy cows (Healthy group) (SCC < 100,000 cells/mL, no obvious signs of the udder and milk, CMT negative); 5 cows with subclinical mastitis (SM group) (200,000 < SCC < 2,000,000 cells/mL, no obvious signs of inflammation of the udder and milk, CMT positive); 5 cows with clinical mastitis (CM group) (SCC > 2,000,000 cells/mL; obvious signs including flakes, clots, or pus in milk, or udder redness, swelling, heat, pain). The basic information on cows, including parity, days in milk, milk yield, udder clinical symptoms, CMT results, milk SCC, and AccuMast results, were displayed in Supplementary File Table S1.

### 2.2. Preparation and Isolation of Milk-Derived EVs

For the isolation of milk-derived EVs, a combination method of acetic acid/ultracentrifugation [9] and density gradient ultracentrifugation [7] was performed in this study. Milk samples were pre-warmed for 10 min at 37 °C, then mixed with acetic acid [milk/acetic acid = 100/5 (vol.)] for 5 min at room temperature. To remove milk fat, somatic cells, and debris, milk samples were centrifuged at 10,000× *g* for 10 min at 4 °C. The Optima XE-90 Ultracentrifuge (Beckman Coulter, Brea, CA, USA) was used in this study. After filtering with a 0.22-μm membrane, the whey was centrifuged with SW32Ti rotor at 150,000× *g* for 70 min at 4 °C. Density gradient ultracentrifugation was performed as described previously [7]. A discontinuous sucrose gradient consisting of 43%, 35%, and 20% solutions of sucrose was prepared in 10 mM Tris, pH 7.5. The exosomal pellets were resuspended with 5 mL PBS and layered on the top slowly. The gradient solutions were centrifuged with SW32Ti rotor (Beckman Coulter, Brea, CA, USA) at 110,000× *g* for 90 min at 4 °C. We collected the exosomal pellets from the 43%/35% interface (an average density of 1.7 g/mL). The exosomal pellets were washed with PBS and centrifuged with SW41Ti rotor at 110,000× *g* for 90 min at 4 °C. The exosomal pellets were resuspended in 200 μL PBS, filtered with a 0.22-µm membrane, and stored at −80 °C until use.

### 2.3. Identification of Milk-Derived EVs

The nanoparticle tracking analysis (NTA) was performed at VivaCellBiosceinces with ZetaView PMX-10 (Particle Metrix, Meerbusch, Germany) to determine the EV’s concentration and size distribution. NTA measurement was taken according to the manufacturer’s instructions. Polystyrene beads 110 nm in size were used to focus the camera and calibrate the instrument. Transmission electron microscopy (TEM) was examined by HT7500 transmission electron microscope (HITACHI, Tokyo, Japan). The isolated vesicles were spotted on Formvar^®^ coated copper grids (Electron Microscopy China, Beijing, China) (200 mesh) and negatively stained with 2% uranyl acetate. We confirmed exosome recovery by western blotting to detect exosome common markers TSG101 and Hsp70 [3]. The vesicles were lysed with RIPA lysis buffer (Sigma-Aldrich, St. Louis, MO, USA) mixed with protease and phosphatase inhibitors (Roche, Branford, CT, USA). Protein concentration was measured by the Pierce^TM^ BCA protein assay kit (Thermo Fisher Scientific, Waltham, MA, USA). The vesicle proteins were separated by SDS-polyacrylamide gel electrophoresis and electrotransferred to a polyvinylidene fluoride membrane using a blotting transfer pack system (BioRad, Hercules, CA, USA). The membranes were incubated with the primary antibodies overnight at 4 °C: rabbit anti-TSG101 (ab125011, Abcam, Shangai, China) at 1/200 dilution and rabbit anti-Hsp70 (ab181606, Abcam, Shangai, China) at 1/200 dilution in 3% BSA-PBS. The membranes were then washed and incubated with the goat anti-rabbit HRP-conjugated antibody (ab205718, Abcam, Shangai, China) at 1/5000 dilution in 3% BSA-PBS for 1 h with rocking at room temperature. Western blotting signals were visualized using a Tanon 5200 chemiluminescence detection system (Tanon, Shanghai, China).

### 2.4. Protein Extraction and Digestion

Exosomes were homogenized in 100 mM Tris-HCl buffer (pH 8.5, 7 M Urea, 1% SDS, 5 mM TCEP). A total of 50 μg protein was reduced with 5 mM TECP at 56 °C for 30 min and alkylated with 20 mM iodoacetamide at room temperature for 30 min in dark. Then, proteins were filtered with a 10 kDa Ultrafiltration device and washed three times with 50 mM TEAB buffer pH 8.0. Then, the protein in ultrafiltration was resuspended in 100 μL digestion buffer composed of 50 mm TEAB buffer. Then, trypsin was added with 1:100 (*w*/*w*), and protein digestion was performed overnight at 37 °C. The peptide was washed twice from ultrafiltration with 1% formic acid and dried using a SpeedVa (Thermo Fisher Scientific, Waltham, MA, USA). Finally, the peptide was resuspended in 0.1% formic acid and 2% acetonitrile for a subsequent nano LC-MS/MS analysis.

### 2.5. LC-MS/MS Analysis

A nano LC-MS/MS analysis was performed using an Orbitrap Fusion Tribrid MS (Thermo Scientific, San Jose, CA, USA) equipped with a nanospray flex ion source and coupled with a Dionex UltiMate 3000 RSLC nano system (Thermo, Sunnyvale, CA, USA). According to Meng et al. [23], peptide samples (2 μL) were injected into the PepMap C18 columns (75 μm × 3 mm, 3 μm) at 6 μL/min for online enrichment and then separated on a PepMap C18 column (2 μm, 75 μm × 250 mm) with 0.1% formic acid as buffer A and 0.1% formic acid in 80% acetonitrile as buffer B at 300 nL/min. The peptides were eluted with the following gradients: 0–5 min, 5–12% B; 5–65 min, 12–38% B; 65–72 min, 38–95% B; 72–80 min, 95% B; 80–81 min, 95–5% B; and 81–95 min, 5% B.

### 2.6. DIA Mode

The mass spectrometer was run under data-independent acquisition (DIA)mode, and automatically switched between MS and MS/MS mode. The survey of full scan MS spectra (350–1200 *m*/*z*) was acquired in the Orbitrap at a resolution of 120,000. The automatic gain control (AGC) target was Custom and the maximum fill time was 50 ms. All precursor ions were entered into the collision cell for fragmentation by higher-energy collision dissociation (HCD); the collision energy was 30 eV. The MS/MS resolution was set at 30,000, The AGC target was Custom, and the maximum fill time was 50 ms. DIA was performed with variable isolation windows; each window overlapped 1 *m*/*z*, and the window number was 80. The FAMIS CV was −45 V.

### 2.7. Raw Data Processing

The raw data files were searched using Maxquant against the *Bos taurus* proteome from the Uniprot database (UP000009136). Mass tolerances for precursor and fragment ions were 10 ppm and 0.02 Da, respectively. The proteins and peptides were filtered with a false discovery rate (FDR) < 1%. The enzyme parameter was limited to semi-tryptic peptides with a maximum miscleavage of 2. Carbamidomethyl (C) of the peptides was set as fixed modifications; oxidation (M) and deamidated (NQ) on the N-terminus of proteins were set as variable modifications.

### 2.8. Bioinformatics Analysis

All statistics and plots were performed using R 4.1, Graphpad 7.0, and the OmicShare tools (https://www.omicshare.com/tools accessed on 07 September 2022); The difference between groups was determined using the Limma package to calculate moderated *t*-test. *p* values were corrected by the Benjamini–Hochberg method. Fold change between two groups was calculated as mean (Mastitis)/mean (Healthy) and expressed on a log2 scale. Differently enriched (DE) proteins were identified as given a Benjamini–Hochberg adjusted *p* (Q value) < 0.05 and a fold change of at least 2. Gene Ontology (GO) analysis was performed based on the Gene Ontology database (http://www.geneontology.org/)(accessed on 02 September 2022); significantly enriched GO terms in DE proteins compared to the genome background were defined by hypergeometric test. The Reactome pathway analysis (https://reactome.org)(accessed on 05 September 2022) used a hypergeometric distribution test to generate a probability score, which was corrected for false discovery rate using the Benjamini–Hochberg method. Significantly enriched GO terms and Reactome pathways were determined at FDR ≤ 0.05, compared with the whole reference genes background.

## 3. Results

### 3.1. Characteristics of Milk-Derived EVs

**Figure 1:**
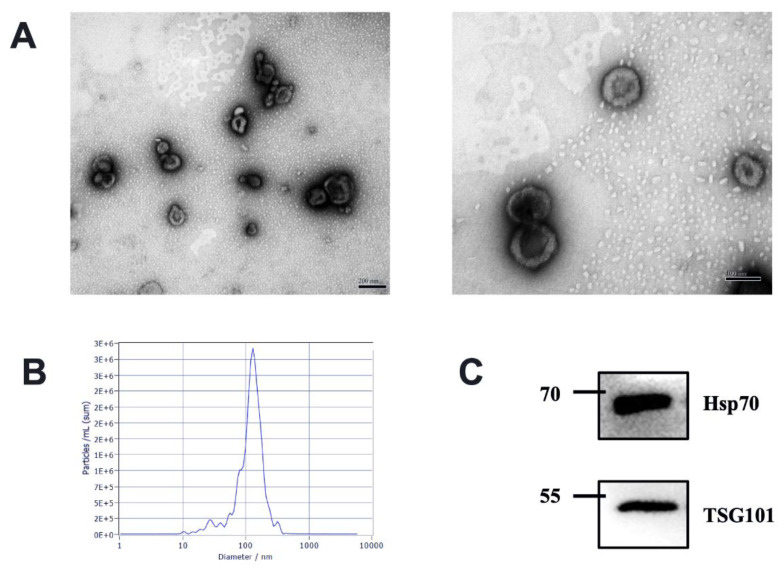
Characterization of EVs from bovine milk (**A**) Transmission electron microscopy observation of milk-derived EVs, left, scale bar size 200 nm; right, scale bar size 100 nm; (**B**) Nanoparticle tracking analysis showing particle size distributions of milk-derived EVs. (**C**) Western blotting of exosome common markers TSG101 and Hsp70 in milk-derived EVs (original western blot figures in Figure S1).

We characterize the isolated milk-derived EVs biophysically through TEM and NTA methods. The milk-derived EVs samples exhibited a mixed population of exosomes, with predominantly intact vesicles, which showed classical exosome-like morphology. We observed EVs characteristic of a spherical shape with a lipid bilayer in the range of 30–150 nm diameter in each sample (Figure 1A). Different health statuses between groups had no effects on the shape of milk EVs. All milk-derived EV samples were also analyzed using NTA; the milk-derived EVs showed a homogenous size distribution with a median diameter of 120 nm (Figure 1B). The presence of exosome markers TSG101 and Hsp70 was confirmed via Western blotting (Figure 1C). Tumor Susceptibility Gene 101 (TSG101) as ESCRT component is involved in multivesicular body formation, and Heat shock protein 70 kDa (Hsp70) as cytosolic protein should be recovered in exosomes [3]. Therefore, our results suggested that the isolated milk-derived EVs subset could be defined as exosomes.

### 3.2. Statistical Analysis of Identified Proteins from Milk-Derived EVs

**Figure 2:**
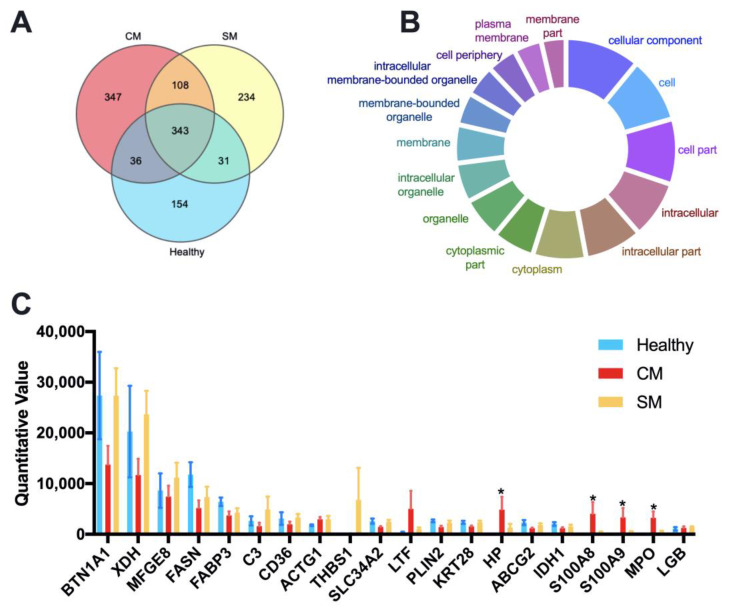
Quantitative proteomic identification of milk-derived EVs proteins. (**A**) Venn diagram illustrating the number of shared and unique proteins among groups; 154, 347, and 234 proteins unique to the Healthy, CM, and SM groups, respectively. (**B**) GeneOntology-cellular component analysis was used to predict the cellular origin of the proteins common to all milk-derived EVs. (**C**) The top 20 abundant proteins based on the average LFQ of all samples. Data are shown as mean ± SEM. Healthy, healthy cows, *n* = 5; CM, subclinical mastitic cows, *n* = 5; SM, subclinical mastitic cows, *n* = 5. * Adjust *p* < 0.05.

We identified and quantitated a total of 1,253 different proteins with an FDR below 1% at the peptide and protein level across all samples. The LFQ (label-free quantitation) results of identified proteins and peptides for each sample are listed in Supplementary File Table S2; the numbers of identified proteins and peptides for each group are listed in Supplementary File Table S3. A Venn diagram identified that 343 proteins were common to all groups, 347 proteins unique to the CM group, 234 proteins unique to the SM group, and 154 proteins unique to the Healthy group (Figure 2A). Cellular component analysis through Gene Ontology tools suggested that the shared proteins were mainly cytoplasmic and membrane proteins (Figure 2B and Table S4). The top 20 abundant proteins were defined by their mean LFQ intensity of all samples, accounting for 76.54%, 59.63%and 73.68% in the Healthy, CM, and SM groups, respectively (Supplementary File Table S5). The four most abundant proteins across all samples were Butyrophilin subfamily 1 member A1 (BTN1AI), Xanthine dehydrogenase (XDH), Lactadherin (MFGE8), Fatty acid synthase (FASN), represented 54.60%, 47.78% and 29.52% of the total abundance in the Healthy, CM, and SM groups, respectively (Figure 2C and Table S5). Among the top 20 abundant proteins, Haptoglobin (HP), Protein S100-A8 (S100A8), Protein S100-A9 (S100A9), and MPO protein (MPO) were significantly more abundant in the CM group than in the Healthy group (adjusted *p* < 0.05).

### 3.3. Mastitis-Associated Alterations in Proteome of Milk-Derived EVs

**Figure 3:**
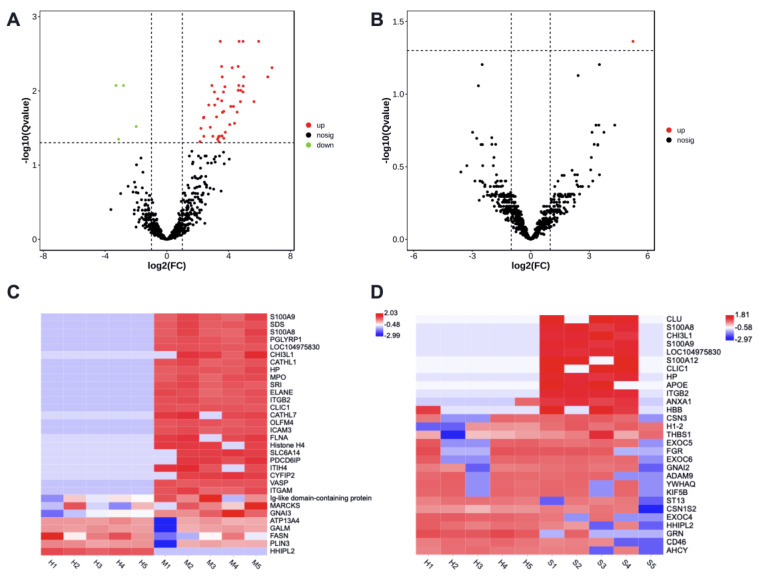
The enrichment of proteins in milk-derived EVs between CM and Healthy group (**A**,**C**), SM and Healthy group (**B**,**D**). Volcano plots show significant proteins with a Q value < 0.05. Heatmaps display the abundance distribution of selected proteins in Table 1 and Table 2; the color key indicates the normalized LFQ intensity per protein. Healthy, healthy cows, *n* = 5; M, subclinical mastitic cows, *n* = 5; S, subclinical mastitic cows, *n* = 5.

There were 53 differently enriched (DE) proteins in milk-derived EVs between CM and Healthy group, including 49 more abundant and 4 less abundant proteins (adjusted *p* < 0.05, Supplementary File Table S6). Protein H1 histone (A7MAZ5) was the only differently abundant in milk-derived EVs between SM and Healthy group (adjusted *p* < 0.05, Supplementary File Table S6). The volcano plot highlighted the significantly increased (red) or decreased (green) proteins associated with mastitis (Figure 3A,B).

Some proteins were only present in one or two samples and could not represent the overall group. Protein H1 histone (A7MAZ5), present in less than three samples in each group, was excluded. Protein was defined as specific only if at least present in three of the five samples and absent in another group. Finally, there were 32 proteins specific to the CM or Healthy group (Table 1) and 29 proteins specific to the SM or Healthy group (Table 2).

The heat maps displayed the distribution of altered proteins among samples (Figure 3C,D), and showed an obvious shift in the milk-derived EVs proteome of CM samples. Compared with the Healthy group, Protein S100-A9, Protein S100-A8, Chitinase-3-like protein 1, Haptoglobin, Integrin beta-2, and Chloride intracellular channel protein 1 were more abundant in the CM group (adjusted *p* < 0.05), but their enrichment in the SM group was not significant. HHIP-likee 2 and Perilipin protein were less abundant in the CM group (adjusted *p* < 0.05), and decreased abundance of HHIP-like 2 was not significant in the SM group. Altogether, these specific milk-derived EVs proteins may thus be used, alone or in combination, as markers to discriminate between the Healthy group and the CM or SM group.

### 3.4. Functional Analysis of DE Proteins in Milk-Derived EVs

**Figure 4:**
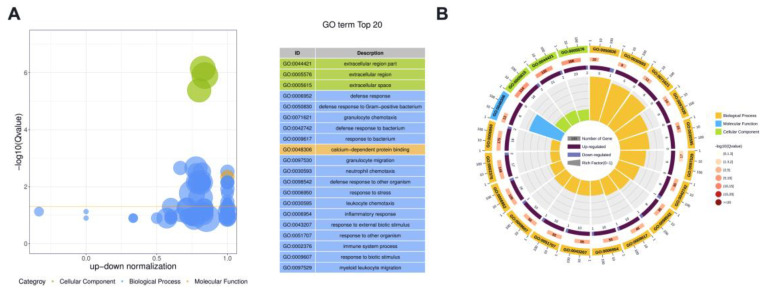
The overview of the top 20 most significant GO terms between CM and Healthy group (Q value < 0.05). (**A**) The bubble plot assigns the z-score to the *x*-axis and the negative logarithm of the adjusted *p*-value to the *y*-axis. The area of circles is proportional to the number of genes assigned to the term, and the color corresponds to the category. (**B**) In the circular barplot from outer to inner, the circle displays three categories; the length represents the total gene number; the color represents Q-value; the area represents the RichFactor value of each category.

We conducted Gene ontology (GO) enrichment analyses to investigate the functions of DE proteins. Because protein H1 histones (A7MAZ5) was the only DE protein in milk-derived EVs between the SM and Healthy group, the role of protein H1 histones (A7MAZ5) was not further analyzed. Figure 4 displays the overview of the significantly enriched GO terms for CM vs. Healthy comparison; details were listed in Supplementary File Table S7. For the CM group, cellular components, including extracellular region part, extracellular region, and extracellular space, were significantly enriched GO terms. Defense response, defense response to Gram-positive bacterium, granulocyte chemotaxis, defense response to bacterium, and response to bacterium were the top five most significant GO terms related to biological processes. Calcium-dependent protein binding was the only significant Go term in the molecular function ontology.

We used the Reactome pathways analysis tool to analyze the pathways potentially impacted by DE proteins between the CM and Healthy group (Table 3). Neutrophil Degranulation was the most significant (FDR < 0.0005) reaction with 16 gene counts, followed by the Innate Immune System with 20 gene counts. The antimicrobial peptides pathway was another significant reaction with 5 gene counts. DE proteins were also associated with RHO GTPases Activate NADPH Oxidases, RHO GTPases Activate WASPs and WAVEs, RHO GTPase Effectors, etc. Details were reported in Supplementary File Table S8. There were 16 proteins, including Granulin precursor, Protein S100-A9, Cytochrome b-245 light chain, Protein S100-A12, C5a anaphylatoxin chemotactic, Peptidoglycan recognition protein 1, Leukocyte elastase inhibitor, Protein S100-A8, Syndecan binding protein, MPO protein, SERPIN domain-containing protein, Cathelicidin-4, Olfactomedin 4, STOM protein, Integrin beta-2, and High-affinity immunoglobulin epsilon receptor subunit gamma, commonly contributed to Neutrophil Degranulation and Innate Immune System. Other 4 proteins, such as Actin-related protein 2/3 complex subunit 1B, Clusterin, Actin-related protein 2/3 complex subunit 2, and Cytoplasmic FMR1-interacting protein, uniquely contributed to the Innate Immune System. The increased pathway antimicrobial peptides were consistent with elevated Cathelicidin-4, Protein S100-A9, Peptidoglycan recognition protein 1(PGLYRP1), Protein S100-A12, and Clusterin—DE proteins found specifically enriched in the CM group.

## 4. Discussion

In this study, we utilized DIA quantitative proteomic approach to investigate the milk-derived EVs proteome from clinical and subclinical mastitis cows. A total of 1253 kinds of proteins were identified and quantified. Identifying a large number of proteins will expand our understanding of milk-derived Ev’s function in mastitis progression [7,16,17,18]. Compared with healthy cows, there were 53 and 1 DE proteins in milk-derived EVs from CM and SM cows, respectively. The enrichment of DE proteins between CM and Healthy group was consistent with elevated GO processes—defense response, defense response to Gram-positive bacterium, granulocyte chemotaxis, also contributed to Reactome pathways—neutrophil degranulation, innate immune system, and antimicrobial peptides in the CM group.

Among the top 20 abundant proteins, protein S100-A9 and protein S100-A8 were more abundant in the CM group than in the Healthy group and showed an increasing trend in the SM group. Consistent with our result, the antimicrobial peptides S100A8, S100A9, and CATHL\H4 were highly expressed in milk exosomes after *Staphylococcus aureus* infection [7,18]. Infection-induced inflammation is one of the primary resources of S100A8/A9 (also known as calprotectin), which is expressed and secreted by immune cells and cells in local lesions [24]. Our data showed that EV protein cargos were altered by mammary infection and inflammation.

S100A8/A9 has dual but related functions in the intracellular and extracellular microenvironment. Intracellular S100A8/A9 complex participates in cytoskeleton regulation, AA metabolism, and defense against pathogens [24,25]. Extracellular S100A8/A9 exerts antibacterial function; stimulates leukocyte recruitment and cytokine secretion [24,25]. Extracellular S100A8/A9 could act as DAMPs (damage associated molecular patterns) via Toll-like receptor 4 (TLR4) for advanced glycation end products (RAGE) [26]. The deficiency of S100A8/A9 in mice could promote the progression of pneumonia caused by *Staphylococcus aureus* infection [27]. The function of S100A8/A9 in milk-derived EVs still needs to be determined.

In this study, AccuMast culture results only indicated the mixed Gram-positive and Gram-negative bacterial infection in the cows with clinical mastitis. We could not elucidate associations between specific exosome proteins and etiological agents based on our data. Combining the 16S rDNA sequencing method to collect the microbiological data and perform an extra bioinformatics analysis using a database with *S. aureus* proteins will provide more significance.

Sprenkeler et al. observed that S100A8/A9 is highly present in the cytoplasmic fraction of neutrophils and was released upon the formation of neutrophil extracellular traps (NETs) [28]. NETs are extracellular structures composed of granule and nuclear neutrophil constituents [28]. Recently, NETs were also found in bovine mastitis, where neutrophil phagocytosis and oxidative burst are hampered by milk fat and proteins [29].

EVs contain S100A8/A9, which can be transferred to another cell and may play a role in that new environment. Exosomes with S100A9 cargo can significantly up-regulate the expression of several pro-inflammatory factors and chemokines and promote the activation of the NF-κB pathway [30,31]. As EVs cargo, S100A8/A9 enrichment may be attributed to the formation of NETs, which widely occur in clinical mastitis for capturing and killing invading pathogens. Therefore, S100A8/A9 contained in milk-derived EVs may participate in pro-inflammatory factors delivery and amplify the activation of the NF-κB pathway [30,31].

For the CM group, GO analysis indicated that the DE proteins in milk-derived EVs were predicted to defense response, defense response to Gram-positive bacterium, and granulocyte chemotaxis. Reactome analysis showed that DE proteins in milk-EVs may contribute to neutrophil degranulation, innate immune system, and antimicrobial peptides in CM cows. Neutrophil degranulation is a double-edged sword in the immune response, which can release various antimicrobial and cytotoxic proteins to participate in the innate immune response, but excessive degranulation can damage host tissue [32]. S100A8, S100A9, and S100A12 are known as potent neutrophil activators, which were also more abundant in the milk-derived EVs from CM cows than in healthy cows in our study. The S100A8/A9–AA complex internalized by infiltrated cells at inflammatory foci could induce neutrophil degranulation via the synthesis of inflammatory mediators, such as leukotriene B4 [33]. EVs play essential roles in intercellular communication. The transferration of S100A8-riched EVs to acceptor cells orchestrates the activation of neutrophil degranulation, thus, can contribute to the innate immune response of clinical mastitis.

Reactome pathway analysis also showed that RHO GTPases Activate NADPH Oxidases, and RHO GTPase Effectors were significant reactions in CM cows. As conserved molecular switches, RHO GTPases can regulate actin dynamics, gene transcription, cell cycle progression, and cell adhesion [34]. Consistent with the previous study, rearrangement of the actin-cytoskeleton through Rho GTPase-regulated pathways was one of the *S. aureus* infection-specific features [35]. The (NADPH) oxidases are major sources of reactive oxygen species (ROS), which are essential in killing neutrophil microorganisms. NADPH oxidase activation and ROS production were enhanced by S100A8/A9 overexpression in epithelial cell lines [36]. S100A8 and S100A9 are expressed in epithelia under specific conditions; they may be secreted by bovine mammary epithelial cells into the milk from CM cows. Thus, milk-derived EVs in CM cows might be involved in mammary pathogenesis by enhancing NADPH oxidase activation or RHO GTPase Effectors, then modulating downstream signaling.

Reactome pathway analysis revealed Cathelicidin-4, Protein S100-A9, Peptidoglycan recognition protein 1, Protein S100-A12, and Clusterin in milk-derived EVs engaged in the significant reaction antimicrobial peptides (AMPs) in CM cows. Indeed, Cathelicidin-4, Protein S100-A9, Peptidoglycan recognition protein 1, and Protein S100-A12 are secreted antimicrobial and proinflammatory proteins. Cathelicidins establish an antimicrobial barrier at epithelial interfaces [37]. PGLYRP1 is abundant in polymorphonuclear leukocyte granules and is released on degranulation to counteract microbial infections [38,39]. Intriguingly, antimicrobial defense by exosomal shuttling of antimicrobial peptides becomes a new arm of mucosal immunity [40].

Due to the limited data in our study, it is difficult to identify more DE proteins associated with subclinical mastitis. The increasing trend of S100A9 and S100A8 in SM was instructive information and required further validation. Milk-derived EVs may play crucial roles in cell-to-cell communication because of their ability to carry AMPs, including Cathelicidin-4, Protein S100-A9, Peptidoglycan recognition protein 1, and Protein S100-A12. Instead of directly secreting AMPs, milk-derived EVs into the mammary duct could be an effective way to reduce cytotoxicity and keep antimicrobial activity. Therefore, our findings may provide attractive ideas for engineering new antimicrobials in animals and humans.

Whether these DE proteins in milk-derived EVs participate in or are a consequence of mammary inflammation is uncertain. Previous studies have explored milk-derived Ev’s function in mastitis and colitis [41,42]. For instance, yak and cow milk exosomes partially exerted a protective effect on LPS-induced colitis by inhibiting PI3K/AKT pathway activation [42]. However, exosome secretion significantly promoted inflammatory cell infiltration in the progression of plasma cell mastitis via the PI3K/Akt/mTOR signaling pathway [41]. Therefore, the role of milk-derived EVs or neutrophil-derived EVs in mastitis therapy has not been fully explored. Further functional assays in vitro and in vivo are necessary to support this hypothesis.

## 5. Conclusions

In this study, we have comprehensively analyzed the proteome of milk-derived EVs from CM/SM and healthy cows. Our data indicated that mammary infection and inflammation altered milk-derived EV protein cargos. Protein S100-A9, Protein S100-A8, Chitinase-3-like protein 1, Haptoglobin, Integrin beta-2, and Chloride intracellular channel protein 1 were more abundant in milk-derived EVs for clinical mastitis. The data obtained in this study expanded the repertoire of bovine milk-derived proteins and conduced more attention to the function. The AMPs contained in milk-derived EVs may inflict research interest for engineering new antimicrobials.

## Figures and Tables

**Table 1:** Statistic analysis of the selected proteins in milk-derived EVs between the CM and Healthy group by moderated *t*-test. Log FC, Fold change calculated as CM/Healthy and expressed on a log2 scale. Adjust *p*, corrected by the Benjamini–Hochberg method.

Uniport IDs	GeneNames	Protein Names	logFC	*p* Value	Adjust *p*
F1MHS5	S100A9	Protein S100-A9	6.81	0.000	0.005
A0A3Q1LTZ6	SDS	L-serine dehydratase/L-threonine deaminase	6.54	0.000	0.006
P28782	S100A8	Protein S100-A8	5.94	0.000	0.002
Q8SPP7	PGLYRP1	Peptidoglycan recognition protein 1	4.94	0.000	0.009
F1N1Z8	LOC104975830	Uncharacterized protein	4.93	0.000	0.010
A0A3Q1MT29	CHI3L1	Chitinase-3-like protein 1	4.76	0.000	0.014
P22226	CATHL1	Cathelicidin-1	4.66	0.000	0.008
Q2TBU0	HP	Haptoglobin	4.62	0.000	0.005
A6QPT4	MPO	MPO protein	4.23	0.000	0.005
Q0IIA3	SRI	Sorcin	3.76	0.000	0.009
A6QPP7	ELANE	ELA2 protein	3.71	0.008	0.096
P32592	ITGB2	Integrin beta-2	3.54	0.000	0.005
Q5E9B7	CLIC1	Chloride intracellular channel protein 1	3.46	0.000	0.002
P56425	CATHL7	Cathelicidin-7	3.37	0.002	0.041
F1MVJ8	OLFM4	Olfactomedin 4	3.29	0.000	0.013
F1MQF0	ICAM3	Intercellular adhesion molecule 3	3.22	0.001	0.022
F1N169	FLNA	Filamin A	3.15	0.005	0.077
A0A3Q1M1Z1	Histone H4	Histone H4	3.10	0.001	0.016
A5D7H9	SLC6A14	Transporter	2.88	0.008	0.094
E1BKM4	PDCD6IP	Programmed cell death 6 interacting protein	2.71	0.000	0.016
A0A3Q1MA31	ITIH4	Inter-alpha-trypsin inhibitor heavy chain H4	2.55	0.005	0.075
F1MX60	CYFIP2	Cytoplasmic FMR1-interacting protein	2.39	0.001	0.023
Q2TA49	VASP	Vasodilator-stimulated phosphoprotein	1.85	0.014	0.148
A0A3Q1M1Z4	Ig-like	Ig-like domain-containing protein	1.69	0.046	0.296
P12624	MARCKS	Myristoylated alanine-rich C-kinase substrate	1.66	0.008	0.096
G3MYD9	ITGAM	Integrin subunit alpha M	1.64	0.032	0.241
Q3ZCA7	GNAI3	G protein subunit alpha i3	1.36	0.020	0.186
F1N272	ATP13A4	Cation-transporting ATPase	−1.11	0.036	0.256
Q5EA79	GALM	Aldose 1-epimerase	−1.18	0.047	0.296
F1N647	FASN	Fatty acid synthase	−1.32	0.030	0.237
Q3SX32	PLIN3	Perilipin	−1.99	0.001	0.030
E1BGX8	HHIPL2	HHIP like 2	−2.81	0.000	0.008

**Table 2:** Statistic analysis of the selected proteins in milk-derived EVs between the SM and Healthy group by moderated *t*-test. Log FC, Fold change calculated as SM/Healthy and expressed on a log2 scale. Adjust *p*, corrected by the Benjamini–Hochberg method.

Uniport IDs	Gene Names	Protein Names	logFC	*p* Value	Adjust *p*
P17697	CLU	Clusterin	3.75	0.002	0.183
P28782	S100A8	Protein S100-A8	3.49	0.002	0.163
A0A3Q1MT29	CHI3L1	Chitinase-3-like protein 1	3.44	0.006	0.225
F1MHS5	S100A9	Protein S100-A9	3.18	0.021	0.396
F1N1Z8	LOC104975830	Uncharacterized protein	3.07	0.011	0.332
P79105	S100A12	Protein S100-A12	2.97	0.034	0.432
Q5E9B7	CLIC1	Chloride intracellular channel protein 1	2.43	0.000	0.074
Q2TBU0	HP	Haptoglobin	2.26	0.014	0.359
A0A3Q1LPF0	APOE	Apolipoprotein E	1.93	0.049	0.488
P32592	ITGB2	Integrin beta-2	1.71	0.015	0.363
F1N650	ANXA1	Annexin	2.19	0.046	0.488
P02070	HBB	Hemoglobin subunit beta	2.51	0.045	0.488
A0A3Q1M5U9	CSN3	Kappa-casein	2.57	0.034	0.432
P02253	H1-2	Histone H1.2	3.11	0.007	0.273
F1N3A1	THBS1	Thrombospondin-1	3.44	0.005	0.222
F1MC71	EXOC5	Exocyst complex component 5	−1.28	0.020	0.396
A5PKG9	FGR	Tyrosine-protein kinase	−1.76	0.040	0.471
A0A3Q1M4P7	EXOC6	Exocyst complex component	−2.22	0.039	0.471
A7MBH9	GNAI2	G protein subunit alpha i2	1.61	0.015	0.363
F1MZJ5	ADAM9	ADAM metallopeptidase domain 9	−1.03	0.041	0.474
Q3SZI4	YWHAQ	14-3-3 protein theta	−1.79	0.043	0.482
F1N1G7	KIF5B	Kinesin-like protein	−1.82	0.049	0.488
A7E3S8	ST13	Heat shock 70kD protein binding protein	−1.45	0.028	0.429
P02663	CSN1S2	Alpha-S2-casein	1.40	0.022	0.397
A6QLD1	EXOC4	EXOC4 protein	−1.80	0.004	0.222
E1BGX8	HHIPL2	HHIP like 2	−2.00	0.003	0.199
E1BHY6	GRN	Granulin precursor	−2.07	0.032	0.432
A0A3Q1LW07	CD46	Membrane cofactor protein	−2.49	0.000	0.063
Q3MHL4	AHCY	Adenosylhomocysteinase	−3.28	0.009	0.311

**Table 3:** Reactome pathways analysis of DE proteins in milk-derive EVs between the CM and Healthy group.

Pathway Name	Entities					Reactions		
	Found	Total	Ratio	*p* Value	FDR	Found	Total	Ratio
Neutrophil degranulation	16	486	0.056	0.00000002	0.000004	9	10	0.001
Innate Immune System	20	901	0.104	0.00000014	0.000015	54	429	0.054
Antimicrobial peptides	5	56	0.006	0.00002490	0.001790	6	38	0.005
Immune System	21	1584	0.183	0.00022800	0.012300	56	1037	0.131
RHO GTPases Activate NADPH Oxidases	3	22	0.003	0.00034800	0.015000	9	14	0.002
Metal sequestration by antimicrobial proteins	2	7	0.001	0.00087400	0.029000	2	5	0.001
RHO GTPases Activate WASPs and WAVEs	3	31	0.004	0.00093600	0.029000	6	7	0.001
RHO GTPase Effectors	7	287	0.033	0.00176000	0.047600	22	88	0.011
Regulation of actin dynamics for phagocytic cup formation	3	53	0.006	0.00426000	0.102000	7	18	0.002
Regulation of TLR by endogenous ligand	2	17	0.002	0.00496000	0.104000	1	11	0.001
EPH-Ephrin signaling	3	65	0.007	0.00746000	0.142000	2	32	0.004
Fcgamma receptor (FCGR) dependent phagocytosis	3	67	0.008	0.00810000	0.146000	7	29	0.004
Toll-like Receptor Cascades	4	140	0.016	0.01060000	0.165000	3	116	0.015
Neurofascin interactions	1	2	0	0.01220000	0.165000	1	1	0
Events associated with phagocytolytic activity of PMN cells	1	2	0	0.01220000	0.165000	4	5	0.001
RIPK1-mediated regulated necrosis	2	29	0.003	0.01380000	0.165000	2	22	0.003
Regulation of necroptotic cell death	2	29	0.003	0.01380000	0.165000	1	16	0.002
EPHB-mediated forward signaling	2	31	0.004	0.01560000	0.184000	1	11	0.001
Signaling by Rho GTPases, Miro GTPases and RHOBTB3	9	653	0.075	0.01670000	0.184000	30	176	0.022
ROS and RNS production in phagocytes	2	37	0.004	0.02170000	0.217000	5	10	0.001

ratio, found/total. FDR, False Discovery Rate.

## Data Availability

The proteome data in this study have been deposited in the Integrated proteome resources (IPX0004384001).

## Supplementary Materials

The following supporting information can be downloaded at: https://www.mdpi.com/article/10.3390/ani13010171/s1, Figure S1: Original western blot figures for figure 1C; Table S1: The basic information of cows included in this study; Table S2: The total indentified proteins from milk-derived EVS by DIA proteomic method in this study; Table S3: The number of indentified proteins and peptides for each group; TableS4: Top 20 of the most enriched GO terms in milk-derived Evs through GeneOntology-cellular component analysis; Table S5: Top 20 of the most enriched proteins in milk-derived Evs based on the LFQ normalized for each sample; Table S6: List of significantly different proteins in milk-derived EVs by CM and SM comparison with Healthy group; Table S7: The significantly enriched GO terms of differently abundant proteins in milk-derived EVs by CM comparison with Healthy group; Table S8: The DE proteins involved in significant Reactome pathways in milk-derived EVs by CM comparison with Healthy group.

## References

[1] Kalluri R., LeBleu V.S. (2020). The Biology, Function, and Biomedical Applications of Exosomes. Science.

[2] Mathieu M., Martin-Jaular L., Lavieu G., Théry C. (2019). Specificities of Secretion and Uptake of Exosomes and Other Extracellular Vesicles for Cell-to-Cell Communication. Nat. Cell Biol..

[3] Théry C., Witwer K.W., Aikawa E., Alcaraz M.J., Anderson J.D., Andriantsitohaina R., Antoniou A., Arab T., Archer F., Atkin-Smith G.K. (2018). Minimal Information for Studies of Extracellular Vesicles 2018 (MISEV2018): A Position Statement of the International Society for Extracellular Vesicles and Update of the MISEV2014 Guidelines. J. Extracell. Vesicles.

[4] Kowal J., Arras G., Colombo M., Jouve M., Morath J.P., Primdal-Bengtson B., Dingli F., Loew D., Tkach M., Théry C. (2016). Proteomic Comparison Defines Novel Markers to Characterize Heterogeneous Populations of Extracellular Vesicle Subtypes. Proc. Natl. Acad. Sci. USA.

[5] Lässer C., Alikhani V.S., Ekström K., Eldh M., Paredes P.T., Bossios A., Sjöstrand M., Gabrielsson S., Lötvall J., Valadi H. (2011). Human Saliva, Plasma and Breast Milk Exosomes Contain RNA: Uptake by Macrophages. J. Transl. Med..

[6] Foster B.P., Balassa T., Benen T.D., Dominovic M., Elmadjian G.K., Florova V., Fransolet M.D., Kestlerova A., Kmiecik G., Kostadinova I.A. (2016). Extracellular Vesicles in Blood, Milk and Body Fluids of the Female and Male Urogenital Tract and with Special Regard to Reproduction. Crit. Rev. Clin. Lab. Sci..

[7] Reinhardt T.A., Lippolis J.D., Nonnecke B.J., Sacco R.E. (2012). Bovine Milk Exosome Proteome. J. Proteom..

[8] Wolf T., Baier S.R., Zempleni J. (2015). The Intestinal Transport of Bovine Milk Exosomes Is Mediated by Endocytosis in Human Colon Carcinoma Caco-2 Cells and Rat Small Intestinal IEC-6 Cells. J. Nutr..

[9] Somiya M., Yoshioka Y., Ochiya T. (2018). Biocompatibility of Highly Purified Bovine Milk-Derived Extracellular Vesicles. J. Extracell. Vesicles.

[10] Feng X., Chen X., Zheng X., Zhu H., Qi Q., Liu S., Zhang H., Che J. (2021). Latest Trend of Milk Derived Exosomes: Cargos, Functions, and Applications. Front. Nutr..

[11] Zempleni J., Sukreet S., Zhou F., Wu D., Mutai E. (2018). Milk-Derived Exosomes and Metabolic Regulation. Annu. Rev. Anim. Biosci..

[12] Ross M., Atalla H., Karrow N., Mallard B.A. (2021). The Bioactivity of Colostrum and Milk Exosomes of High, Average, and Low Immune Responder Cows on Human Intestinal Epithelial Cells. J. Dairy Sci..

[13] Ruegg P.L. (2017). A 100-Year Review: Mastitis Detection, Management, and Prevention. J. Dairy Sci..

[14] Martins L., Barcelos M.M., Cue R.I., Anderson K.L., Dos Santos M.V., Gonçalves J.L. (2020). Chronic Subclinical Mastitis Reduces Milk and Components Yield at the Cow Level. J. Dairy Res..

[15] Busanello M., Rossi R.S., Cassoli L.D., Pantoja J.C.F., Machado P.F. (2017). Estimation of Prevalence and Incidence of Subclinical Mastitis in a Large Population of Brazilian Dairy Herds. J. Dairy Sci..

[16] Samuel M., Chisanga D., Liem M., Keerthikumar S., Anand S., Ang C.-S., Adda C.G., Versteegen E., Jois M., Mathivanan S. (2017). Bovine Milk-Derived Exosomes from Colostrum Are Enriched with Proteins Implicated in Immune Response and Growth. Sci. Rep..

[17] Yang M., Song D., Cao X., Wu R., Liu B., Ye W., Wu J., Yue X. (2017). Comparative Proteomic Analysis of Milk-Derived Exosomes in Human and Bovine Colostrum and Mature Milk Samples by ITRAQ-Coupled LC-MS/MS. Food Res. Int..

[18] Reinhardt T.A., Sacco R.E., Nonnecke B.J., Lippolis J.D. (2013). Bovine Milk Proteome: Quantitative Changes in Normal Milk Exosomes, Milk Fat Globule Membranes and Whey Proteomes Resulting from *Staphylococcus Aureus* Mastitis. J. Proteom..

[19] Cai M., He H., Jia X., Chen S., Wang J., Shi Y., Liu B., Xiao W., Lai S. (2018). Genome-Wide MicroRNA Profiling of Bovine Milk-Derived Exosomes Infected with *Staphylococcus Aureus*. Cell Stress Chaperones.

[20] Sun J., Aswath K., Schroeder S.G., Lippolis J.D., Reinhardt T.A., Sonstegard T.S. (2015). MicroRNA Expression Profiles of Bovine Milk Exosomes in Response to *Staphylococcus Aureus* Infection. BMC Genom..

[21] Ma S., Tong C., Ibeagha-Awemu E.M., Zhao X. (2019). Identification and Characterization of Differentially Expressed Exosomal MicroRNAs in Bovine Milk Infected with *Staphylococcus Aureus*. BMC Genom..

[22] Saenz-de-Juano M.D., Silvestrelli G., Bauersachs S., Ulbrich S.E. (2022). Determining Extracellular Vesicles Properties and MiRNA Cargo Variability in Bovine Milk from Healthy Cows and Cows Undergoing Subclinical Mastitis. BMC Genom..

[23] Meng Q., Chen L., Xiong B., Kang B., Zhang P., Tang S., Han H., Shen W., Feng X., Feng S. (2021). Single-Cell Transcriptome Sequencing and Proteomics Reveal Neonatal Ileum Dynamic Developmental Potentials. Msystems.

[24] Wang S., Song R., Wang Z., Jing Z., Wang S., Ma J. (2018). S100A8/A9 in Inflammation. Front. Immunol..

[25] Xia C., Braunstein Z., Toomey A.C., Zhong J., Rao X. (2018). S100 Proteins As an Important Regulator of Macrophage Inflammation. Front. Immunol..

[26] Vogl T., Gharibyan A.L., Morozova-Roche L.A. (2012). Pro-Inflammatory S100A8 and S100A9 Proteins: Self-Assembly into Multifunctional Native and Amyloid Complexes. Int. J. Mol. Sci..

[27] Achouiti A., Vogl T., Van der Meer A.J., Stroo I., Florquin S., de Boer O.J., Roth J., Zeerleder S., van′t Veer C., de Vos A.F. (2015). Myeloid-Related Protein-14 Deficiency Promotes Inflammation in Staphylococcal Pneumonia. Eur. Respir. J..

[28] Sprenkeler E.G., Zandstra J., van Kleef N.D., Goetschalckx I., Verstegen B., Aarts C.E., Janssen H., Tool A.T., van Mierlo G., van Bruggen R. (2022). S100A8/A9 Is a Marker for the Release of Neutrophil Extracellular Traps and Induces Neutrophil Activation. Cells.

[29] Wartha F., Beiter K., Normark S., Henriques-Normark B. (2007). Neutrophil Extracellular Traps: Casting the NET over Pathogenesis. Curr. Opin. Microbiol..

[30] Prieto D., Sotelo N., Seija N., Sernbo S., Abreu C., Durán R., Gil M., Sicco E., Irigoin V., Oliver C. (2017). S100-A9 Protein in Exosomes from Chronic Lymphocytic Leukemia Cells Promotes NF-ΚB Activity during Disease Progression. Blood.

[31] Li H., Huang X., Chang X., Yao J., He Q., Shen Z., Ji Y., Wang K. (2020). S100-A9 Protein in Exosomes Derived from Follicular Fluid Promotes Inflammation via Activation of NF-κB Pathway in Polycystic Ovary Syndrome. J. Cell. Mol. Med..

[32] Lacy P. (2006). Mechanisms of Degranulation in Neutrophils. Allergy Asthma Clin. Immunol..

[33] Han C., Huang H., Hu M., Wang Q., Gao Y., Liu Y. (2007). Time-Dependent Expression of Leukotriene B4 Receptors in Rat Collagen-Induced Arthritis. Prostaglandins Other Lipid Mediat..

[34] Bishop A.L., Hall A. (2000). Rho GTPases and Their Effector Proteins. Biochem. J..

[35] Günther J., Petzl W., Bauer I., Ponsuksili S., Zerbe H., Schuberth H.-J., Brunner R.M., Seyfert H.-M. (2017). Differentiating Staphylococcus Aureus from Escherichia Coli Mastitis: S. Aureus Triggers Unbalanced Immune-Dampening and Host Cell Invasion Immediately after Udder Infection. Sci. Rep..

[36] Benedyk M., Sopalla C., Nacken W., Bode G., Melkonyan H., Banfi B., Kerkhoff C. (2007). HaCaT Keratinocytes Overexpressing the S100 Proteins S100A8 and S100A9 Show Increased NADPH Oxidase and NF-ΚB Activities. J. Investig. Dermatol..

[37] Nardo A.D., Braff M.H., Taylor K.R., Na C., Granstein R.D., McInturff J.E., Krutzik S., Modlin R.L., Gallo R.L. (2007). Cathelicidin Antimicrobial Peptides Block Dendritic Cell TLR4 Activation and Allergic Contact Sensitization. J. Immunol..

[38] Dziarski R., Platt K.A., Gelius E., Steiner H., Gupta D. (2003). Defect in Neutrophil Killing and Increased Susceptibility to Infection with Nonpathogenic Gram-positive Bacteria in Peptidoglycan Recognition Protein-S (PGRP-S)–Deficient Mice. Blood.

[39] Rathnayake N., Gustafsson A., Sorsa T., Norhammar A., Bostanci N., PAROKRANK Steering Committee (2022). Association of Peptidoglycan Recognition Protein 1 to Post-myocardial Infarction and Periodontal Inflammation: A Subgroup Report from the PAROKRANK (Periodontal Disease and the Relation to Myocardial Infarction) Study. J. Periodontol..

[40] Hu G., Gong A.-Y., Roth A.L., Huang B.Q., Ward H.D., Zhu G., LaRusso N.F., Hanson N.D., Chen X.-M. (2013). Release of Luminal Exosomes Contributes to TLR4-Mediated Epithelial Antimicrobial Defense. PLoS Pathog..

[41] Wang X., Han Y., Liu J., Zhang Y., Cheng K., Guo J., Guo Q., Liu S., Sun H., Hua Y. (2019). Exosomes Play an Important Role in the Progression of Plasma Cell Mastitis via the PI3K-Akt-MTOR Signaling Pathway. Mediat. Inflamm..

[42] Gao H.N., Hu H., Wen P.C., Lian S., Xie X.L., Song H.L., Yang Z.N., Ren F.Z. (2021). Yak Milk-Derived Exosomes Alleviate Lipopolysaccharide-Induced Intestinal Inflammation by Inhibiting PI3K/AKT/C3 Pathway Activation. J. Dairy Sci..

