# Supplementary figures and images for: Comparative Proteomic Analysis of Milk-Derived Extracellular Vesicles from Dairy Cows with Clinical and Subclinical Mastitis
Source: Animals (Basel). 2023 Jan 1;13(1):171. doi: 10.3390/ani13010171 (PMC9818007; doi:10.3390/ani13010171)

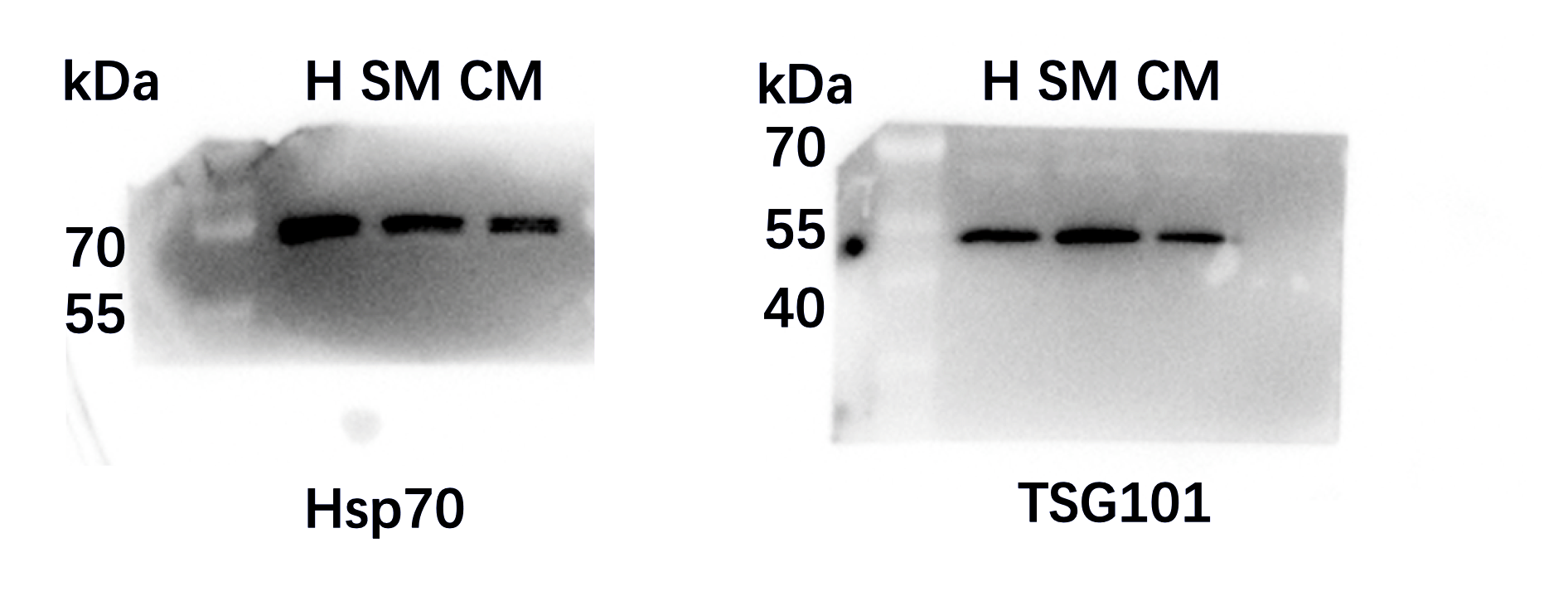

Supplement: Supplementary file 1 [file animals-13-00171-s001.zip › animals-1987791-supplementary/Figure S1.tiff]
